# Association between leptin and NAFLD: a two-sample Mendelian randomization study

**DOI:** 10.1186/s40001-023-01147-x

**Published:** 2023-07-03

**Authors:** Ziwei Guo, Hongbo Du, Yi Guo, Qian Jin, Ruijia Liu, Zhangjun Yun, Jiaxin Zhang, Xiaoke Li, Yong’an Ye

**Affiliations:** 1 Dongzhimen Hospital, Beijing University of Chinese Medicine, Beijing, 100700 China; 2 Beijing University of Chinese Medicine, Beijing, 100102 China; 3 Institute of Liver Diseases, Beijing University of Chinese Medicine, Beijing, China

**Keywords:** Nonalcoholic fatty liver disease, Leptin, Causal effect, Two-sample Mendelian randomization

## Abstract

**Background:**

The etiology of nonalcoholic fatty liver disease (NAFLD) involves a complex interaction of genetic and environmental factors. Previous observational studies have revealed that higher leptin levels are related to a lower risk of developing NAFLD, but the causative association remains unknown. We intended to study the causal effect between leptin and NAFLD using the Mendelian randomization (MR) study.

**Methods:**

We performed a two-sample Mendelian randomization (TSMR) analysis using summary GWAS data from leptin (up to 50,321 individuals) and NAFLD (8,434 cases and 770,180 controls) in a European population. Instrumental variables (IVs) that satisfied the three core assumptions of Mendelian randomization were selected. The TSMR analysis was conducted using the inverse variance weighted (IVW) method, MR-Egger regression method, and weighted median (WM) method. To ensure the accuracy and stability of the study results, heterogeneity tests, multiple validity tests, and sensitivity analyses were conducted.

**Results:**

The findings of the TSMR correlation analysis between NAFLD and leptin were as follows: IVW method (odds ratio (OR) 0.6729; 95% confidence interval (95% CI) 0.4907–0.9235; *P* = 0.0142), WM method (OR 0.6549; 95% CI 0.4373–0.9806; *P* = 0.0399), and MR-Egger regression method (*P* = 0.6920). Additionally, the findings of the TSMR correlation analysis between NAFLD and circulating leptin levels adjusted for body mass index (BMI) were as follows: IVW method (OR 0.5876; 95% CI 0.3781–0.9134; *P* = 0.0181), WM method (OR 0.6074; 95% CI 0.4231–0.8721; *P* = 0.0069), and MR-Egger regression method (*P* = 0.8870). It has also been shown that higher levels of leptin are causally linked to a lower risk of developing NAFLD, suggesting that leptin may serve as a protective factor for NAFLD.

**Conclusions:**

Using TSMR analysis and the GWAS database, we investigated the genetic relationship between elevated leptin levels and lowered risk of NAFLD in this study. However, further research is required to understand the underlying mechanisms.

## Background

Over the past two decades, nonalcoholic fatty liver disease (NAFLD) has progressed from a relatively unknown disease to the leading cause of chronic liver disease worldwide [1]. Its global frequency is quickly increasing, reaching up to 25% in developed countries like the United States [2]. NAFLD is a degenerative disease caused by the buildup of intracellular lipid droplets in liver cells, which can induce inflammation, cell death, and even more advanced stages such as nonalcoholic steatohepatitis (NASH) (with or without fibrosis), cirrhosis, and liver cancer [3, 4]. Currently, pharmacological options for NAFLD are limited. Treatment cornerstones are a healthy lifestyle and weight loss. There is still an unmet therapeutic need [5].

NAFLD is bidirectionally associated with components of the metabolic syndrome [6], a cluster of alterations that includes centripetal obesity, decreased HDL cholesterol concentrations, increased triglyceride concentrations, arterial hypertension, and hyperglycemia [7–9]. This syndrome has become one of the epidemics of the twenty-first century. Causative factors include insulin resistance, leptin, lipocalins, microbiota alterations, and epigenetics [10, 11]. Among these, leptin is a molecule secreted primarily from adipose tissues. The circulating levels of leptin are proportional to abrupt changes in percent body fat mass or caloric intake. It is a key regulator of metabolism and energy homeostasis [12]. A potential dual role has been shown between leptin and NAFLD, with leptin possibly exerting an anti-teratogenic effect while also having a pro-inflammatory and pro-fibrotic impact [13–16]. At the same time, observational clinical studies have shown a relationship between persistent hyperleptinemia and the development of steatosis, fibrogenesis, and hepatocellular carcinoma, suggesting that hyperleptinemia is an independent predictor of the presence or development of NAFLD [12, 17, 18]. According to one study, leptin modulated the function of target cells (hepatocytes and macrophages) and controlled their pyroptosis-like cell death via CD8+ T lymphocytes. The interference of leptin and immune cell-related pathways may provide promising strategies for the treatment of NAFLD [19]. However, the relationship between leptin and NAFLD still needs to be clarified.

In epidemiological studies, the presence of confounders dramatically perturbs causality inferences between exposures and outcomes because causality inferences in observational studies are often challenged by potential confounding biases and reverse causality. Additionally, randomized controlled trials (RCTs) have limitations related to ethical issues, observation time, and resources and costs [20]. Mendelian randomization (MR) is an approach that uses genetic variants associated with specific exposures of interest to study the causal effects of modifiable exposures (potential risk factors) on health, social, and economic outcomes [20–24]. In recent years, genome-wide association studies (GWAS) have accumulated millions of data points on associations between genetic variants and complex diseases or phenotypes [25, 26]. Two-sample Mendelian randomization (TSMR) analysis is an optimized extension of the one-sample Mendelian randomization (OSMR) analysis, in which aggregated statistics from published GWAS are used instead of individual-level data, with distinct samples for exposure variables and outcome markers. It allows for the evaluation of the causal impact of exposure factors on outcomes without the need for additional studies, reducing research expenditures and improving the bioinformatic application [23, 27, 28].

To the best of our knowledge, no MR investigations have been conducted to investigate the potential causative link between leptin and NAFLD. In this study, to provide some basis for the prevention and treatment of NAFLD and lower the incidence and disease burden, TSMR analysis was utilized to analyze the possible causal relationship between leptin and NAFLD from a genetic perspective using GWAS summary statistics for leptin and NAFLD with leptin-related gene polymorphisms as instrumental variables (IVs).

## Methods

**Fig. 1:**
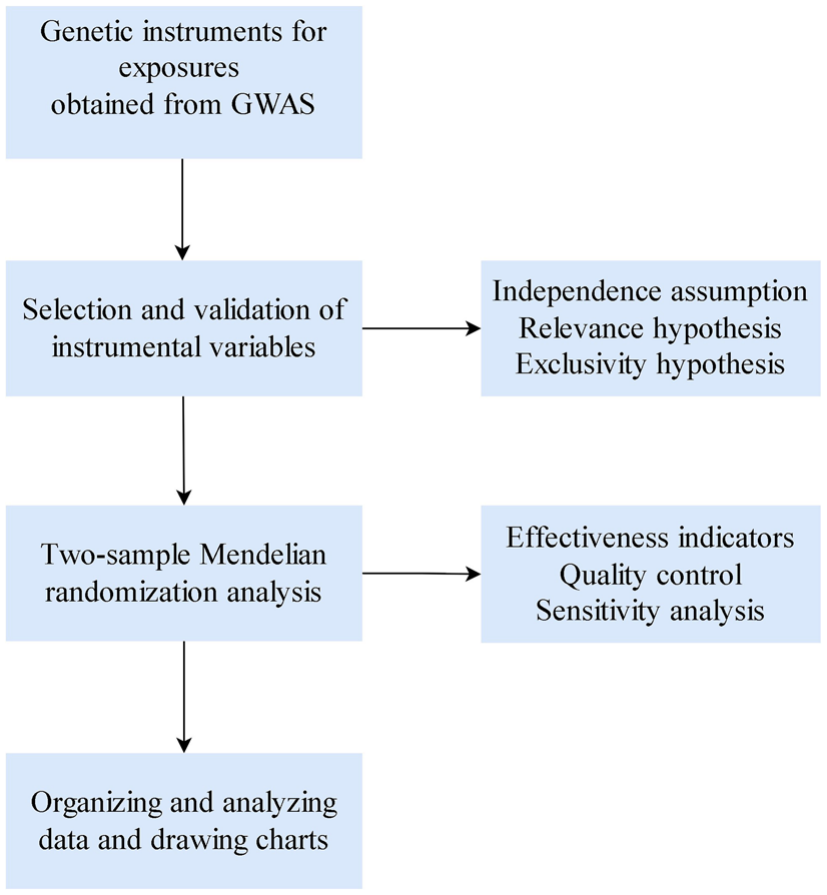
Workflow of this Mendelian randomization study. GWAS, genome-wide association study

We performed a TSMR using publicly available summary-level data from several large-scale cohorts. An overview of this study design is shown in Fig. 1.

### Data type

The TSMR analysis method's data type requires the analysis of data for exposure factors and outcome indicators from two samples. The information required consists of the rs code for single nucleotide polymorphism (SNP), the exposure factor corresponding to the SNP, and the practical value and standard error from the outcome indicator database. Nonessential but useful information includes the frequency of the influencing gene, the name of the gene-coding phenotype, and some information that may be useful in subsequent analyses, such as sample size, number of cases and controls, and chromosomes where SNPs are located. Wherever possible, we also included an indication of the importance of risk genes in exposure factors [29].

### GWAS summary data for leptin

We retrieved genetic data for leptin from the online platform of the Integrated Epidemiology Unit (IEU) Open Genome-Wide Association Studies (GWAS) project (https://gwas.mrcieu.ac.uk/), a database developed by the MRC-IEU at the University of Bristol [30]. It is a collection of the complete GWAS abstract dataset, and data can be downloaded as an open-source file. Two sets of genetic tools were used to reveal the causal relationship between leptin and NFALD. The search codes were "ebi-a-GCST90007310" and " ebi-a-GCST90007322". In this GWAS [31], genetic data on leptin were obtained from a cohort of 35 adults (≥ 18 years), including 57,232 adults of European origin, 4387 of African origin, 2036 of East Asian origin, and 488 of Hispanic origin. Furthermore, because of the phenotypic association between leptin concentration and body mass index (BMI), which was positively associated, this GWAS was corrected for BMI as a confounder between leptin and obesity. To minimize any potential bias induced by the phenotypic association between leptin levels and body mass index, circulating leptin levels were adjusted for BMI using data from this GWAS which included 49,830 sample size. In addition, it included only participants in the European population. Participants were asked to select one of the three categories, "about average," "thinner," or "fuller," to describe their body size at the age of 10 years in comparison to the average. The above two sets of genetic tools were used simultaneously to reveal the causal relationship between leptin and NFALD, increasing the reliability and significant correlation of our study [31].

In addition, in causal inference, we are interested not only in the extent to which exposure affects outcome, but also in the mechanisms or pathways by which exposure affects that outcome [32]. Therefore, to further investigate the potential mechanisms by which genetically determined leptin reduces the risk of NAFLD and to assess whether body composition and adipose tissue can mediate the causal effect of leptin on NAFLD, we further introduced body composition and adipose tissue mass, all of which GWAS data can be found in the IEU (https://gwas.mrcieu.ac.uk/) via ID. Leptin was considered as exposure and body composition and adipose tissue mass such as body fat mass (Visceral adipose tissue volume, Arm fat mass), waist circumference and waist-hip ratio were considered as outcomes to explore potential mediators of the relationship between exposure and outcome. Estimates of IVW were assessed as the main results. *P* < 0.05 was regarded as significant.

### GWAS summary data for NAFLD

GWAS data for NFALD from a genome-wide meta-analysis were based on 4 European cohorts containing 8,434 cases and 770,180 controls. The diagnosis of NAFLD in these 4 cohorts was determined based on the electronic health records of all participants [33]. We download these GWAS data from the GWAS catalog (https://www.ebi.ac.uk/gwas/) and their GWAS Catalog accession number is GCST90011885. More detailed documentation of this GWAS data can be obtained from the original literature. In addition, NAFLD was defined by the use of EHR codes (ICD9: 571.5, ICD9: 571.8, ICD9: 571.9, ICD10: K75.81, ICD10: K76.0 and ICD10: K76.9. Exclusion criteria included, but were not limited to alcohol dependence, alcoholic liver disease, alpha-1 antitrypsin deficiency, Alagille syndrome, liver transplant, cystic fibrosis, hepatitis, abetalipoproteinemia, LCAT deficiency, lipodystrophy, disorders of copper metabolism Reye’s syndrome, inborn errors of metabolism, HELLP syndrome, starvation and acute fatty liver (as suggested by the American Association for the Study of Liver Disease [AASLD]). This study performed a new GWAS for NAFLD in the UK Biobank (data application number 25205). NAFLD diagnosis was established from hospital records (ICD10: K74.0 and K74.2 (hepatic fibrosis), K75.8 (NASH), K76.0 (NAFLD) and ICD10:K76.9 (other specified diseases of the liver). The ICD codes used to define the International Classification of Diseases diagnosis for NFALD can be searched at https://risteys.finngen.fi/.

### Selection and validation of instrumental variables

**Fig. 2:**
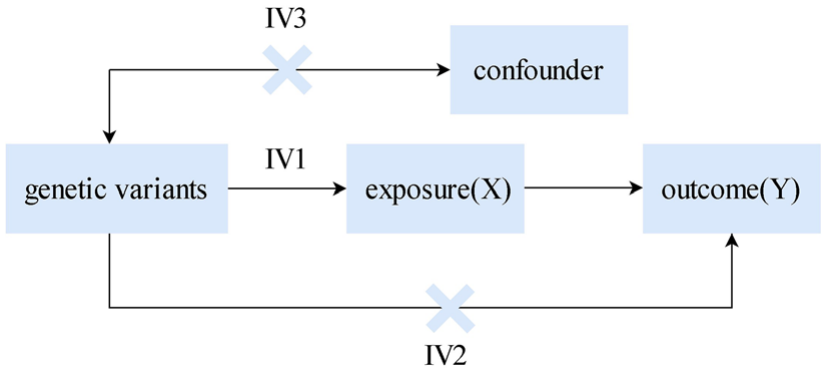
Basic assumptions of Mendelian randomization. IV1: IVs are strongly correlated with exposure. IV2: IVs are independent of outcomes. IV3: IVs are not related to confounding factors

According to the requirements of MR analysis, genetic variants as IVs must satisfy the following three basic assumptions: (I) instrumental variables and exposure factor X have a strong relationship; (II) instrumental variables are not associated with any confounders of the exposure-outcome association; and (III) instrumental variables have no effect on outcome Y, other than perhaps through their association with exposure X [34–36]. Basic assumptions of Mendelian randomization are shown in Fig. 2.

### Statistical analysis

This study used R studio 4.1.1 for statistical analysis, and the TwoSampleMR package is publicly available for download at https://mrcieu.github.io/TwoSampleMR/. First, the effect values and standard errors between IVs and exposure factors and IVs and outcome indicators were obtained by selecting IVs, respectively. Leptin, circulating leptin levels adjusted for BMI, and NAFLD-related GWAS data were imported into R software and assigned to exposure and outcome groups, respectively. To confirm that the effect allele was associated with higher levels of exposure, we harmonized the GWAS data of exposures and outcomes. Next, MR analyses were carried out using various MR methods. Finally, the causal effect values between leptin and NAFLD were estimated using the TSMR method, and quality control and sensitivity analyses were performed. The inverse variance weighted (IVW) method was used as the primary statistical analysis method and supplemented by two additional sensitivity analyses [37], including the weighted median (WM) [38] and MR-Egger methods [39, 40]. The IVW method was used as the primary statistical analysis to estimate the causal effect between exposure factors and outcome indicators. The IVW method requires certainty that all SNPs included meet the three assumptions of IV selection, especially the exclusion assumption, which states that genetic variation influences the outcome indicators only through the exposure factors in the study [41]. This study also used the MR-Egger regression and WM methods to test the stability and reliability of the results. The MR-Egger regression method modifies the IVW method to account for pleiotropy bias [42]. The WM method only requires a minimum of 50% of the weights contributed by genetic variation. When some genetic variations in the analysis are invalid for the IVs, this approach produces consistent results [37].

This study used the odds ratio (OR) as the primary effect indicator, including the 95% confidence interval (CI). Due to data from numerous distinct GWAS cohort studies with potential variances between the studies, such as alternative platforms for gene annotation analysis, additional inclusion and exclusion criteria for cases, and unreliable population sources, the TSMR analysis approach may be heterogeneous and result in biased estimations of causal effects. Cochran's Q test was used in this work to assess the heterogeneity of the IVW and MR-Egger regression [43, 44]. Heterogeneity does not affect the study results if the heterogeneity test result *P* > 0.05 is not statistically significant. For the test of multiplicity, the presence of genetic diversity was tested for causal inference between exposure factors and outcomes according to the exclusion hypothesis. The magnitude of horizontal pleiotropy was expressed as the intercept term in the MR-Egger regression [42], where the closer the intercept was to 0, the smaller the intercept. When the test result for horizontal pleiotropy was *P* > 0.05, there was considered to be none [45]. It was required to run a leave-one-out sensitivity analysis on the data, remove one SNP in turn, and determine the Mendelian randomization analysis effect of the remaining SNPs because there could have been some inevitable random errors while selecting IVs for inclusion [46]. The influence of each SNP on the results can be judged visually by displaying forest plots, and thus, the stability of the TSMR analysis results can be considered. The forest plot can be used to visually determine the effect of each SNP on the results and, therefore, the stability of the TSMR analysis results.

## Results

### Determination of IVs

We extracted the intersection of SNPs as IVs from the gene instrument. To include more SNPs that contributed to circulating leptin levels, a more relaxed threshold (*P* < 5 × 10^−6^) was used, and after removing linkage disequilibrium (LD) (*R*^2^ < 0.01), we retained five eligible SNPs. We then evaluated the remaining SNPs’ power using the F statistics (*F* = beta2/se2) for each SNP and calculate general F statistics for all SNPs. SNPs with less statistical power (rs10938397, rs1121980 and rs780094) were removed (*F* statistics < 10) [47]. Finally, seven SNPs were considered suitable, and the relevant information included the chromosome number, position, β coefficient, Ses, and *P* value. In addition, a total of seven eligible SNPs were obtained from circulating leptin levels that were adjusted for BMI. The information about the above two groups of SNPs is shown in Table 1.

**Table 1:** The genetic instruments used in this study

Trait	SNP	CHR	POS	BETA-exposure	SE-exposure	Pval-exposure	BETA-outcome	SE-outcome	Pval-outcome
Leptin	rs10195252	2	165,513,091	0.0547642	0.382155	3.40E−14	− 0.02402	0.442346	0.1455
	rs1167827	7	75,163,169	0.0338376	0.545837	4.84E−06	− 0.0137983	0.539761	0.4038
	rs62621812	7	127,015,083	− 0.0976946	0.0314287	8.18E−08	0.0114977	0.019881	0.7895
	rs675209	6	7,102,084	0.0358979	0.723037	6.25E−07	− 0.0071326	0.730616	0.6937
	rs713586	2	25,158,008	0.0311325	0.466499	2.45E−06	0.0045135	0.470179	0.781
	rs900399	3	156,798,732	− 0.0330473	0.396124	2.43E−06	0.0330816	0.389662	0.04598
	rs972283	7	130,466,854	− 0.041373	0.521301	1.12E−10	0.0246088	0.560636	0.132
Circulating leptin levels adjusted for BMI	rs1260326	2	27,730,940	0.0477379	0.607045	4.32E−13	− 0.0755351	0.589463	5.98E−06
	rs13389219	2	165,528,876	0.0525424	0.393889	1.13E−13	− 0.0258577	0.435388	0.1189
	rs3799260	6	53,519,605	− 0.0382049	0.818308	3.83E−06	− 0.0275366	0.795229	0.2117
	rs4731702	7	130,433,384	0.0551502	0.483626	6.58E−18	− 0.0305338	0.449304	0.05943
	rs75193761	16	67,233,243	0.0726956	0.0453025	3.45E−06	0.012859	0.030815	0.7553
	rs791600	7	127,865,816	− 0.0626202	0.411084	5.35E–19	0.0149012	0.421471	0.3673
	rs900399	3	156,798,732	− 0.040245	0.39616	9.25E−09	0.0330816	0.389662	0.04598

### Association between genetically predicted NAFLD and leptin

The causal effect between NAFLD and leptin was estimated using the IVW, MR-Egger, and WM methods, as described in the methodology section. The results of the TSMR correlation analysis between NAFLD and leptin were as follows: IVW (OR 0.6729; 95% CI 0.4907–0.9235; *P* = 0.0142), WM method (OR 0.6549; 95% CI 0.4373–0.9806; *P* = 0.0399), and MR-Egger regression method (*P* = 0.6920). It indicates that elevated leptin levels are causally associated with a reduced risk of NAFLD. Also, this study further analyzed the association between NAFLD and circulating leptin levels that were adjusted for BMI. The results of the TSMR correlation analysis were as follows: IVW (OR 0.5876; 95% CI 0.3781–0.9134; *P* = 0.0181), WM method (OR 0.6074; 95% CI 0.4231–0.8721; *P* = 0.0069), and MR-Egger regression method (*P* = 0.8870). It adds to the credibility of the preceding outcomes. The results of the TSMR correlation study are shown in Table 2. In addition, the findings found limited evidence to support a causal relationship between leptin and body composition and adipose tissue, as shown in Table 3.

**Table 2:** TSMR correlation analysis of leptin/Circulating leptin levels adjusted for BMI and NAFLD

Exposure	Outcome	Method	nSNP	Beta	SE	OR (95% CI)	Pval
Leptin	NAFLD	MR Egger	7	− 0.232333254	0.553353711	0.79 (0.26–2.34)	0.69201616
		Weighted median	7	− 0.423262908	0.205995127	0.65 (0.43–0.98)	0.039905775
		Inverse variance weighted	7	− 0.396048774	0.16149831	0.67 (0.49–0.92)	0.014193046
Circulating leptin levels adjusted for BMI	NAFLD	MR Egger	7	0.209529839	1.402200441	1.23 (0.07–19.25)	0.887055213
		Weighted median	7	− 0.498410207	0.184491007	0.60 (0.42–0.87)	0.006901876
		Inverse variance weighted	7	− 0.531548597	0.224986582	0.58 (0.37–0.91)	0.018148268

**Table 3:** TSMR correlation analysis between leptin and body composition and adipose tissue mass

Exposure	ID	Outcome	Pval	OR (95% CI)
Leptin	ebi-a-GCST90016671	Visceral adipose tissue volume	0.1695895	1.32 (0.88–1.97)
Leptin	ukb-b-6704	Arm fat mass (right)	0.05145062	1.82 (0.99–3.33)
Leptin	ukb-b-8338	Arm fat mass (left)	0.053347628	1.83 (0.99–3.38)
Leptin	ukb-b-9405	Waist circumference	0.178025885	1.55 (0.81–2.93)
Leptin	ieu-a-73	Waist-to-hip ratio	0.666751872	1.17 (0.57–2.39)

### Heterogeneity analysis and Pluripotency analysis

**Fig. 3:**
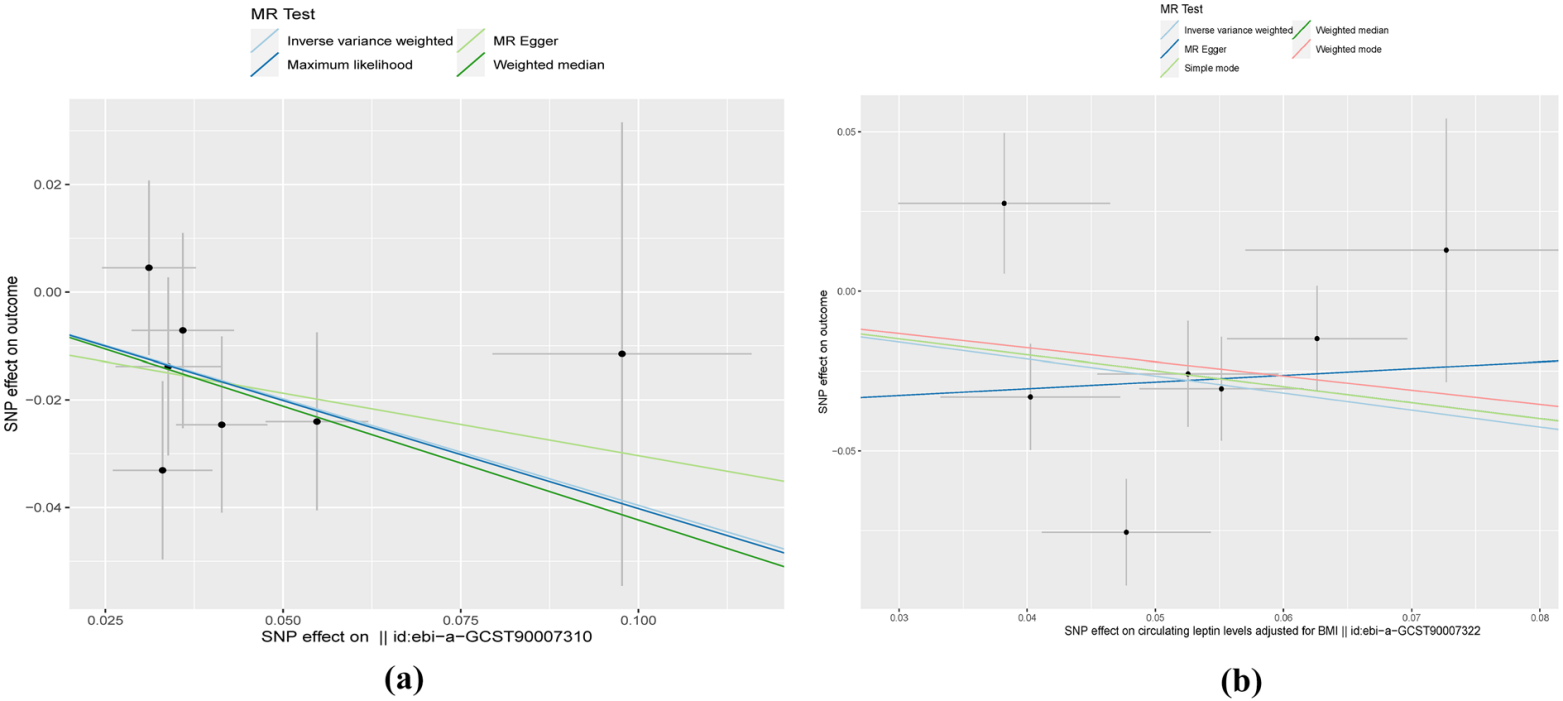
TSMR analysis. The intercept estimate can be interpreted as an estimate of the average pleiotropy of all SNPs, and the slope coefficient estimates the causal effect’s bias. **a** Leptin; **b** circulating leptin levels adjusted for BMI

Heterogeneity tests between NAFLD and leptin genetic variants using Cochran's Q revealed the following: IVW (*P* = 0.762) and MR Egger (*P* = 0.659). There was no heterogeneity, hence the fixed-effects model was preferred for MR analysis. The heterogeneity and pluripotency analysis results are shown in Table 4. Five methods were used to evaluate the results of MR analysis, and scatter plots were generated (Fig. 3). Among them, MR-Egger was used to measure the pluripotency of IVs. There was no statistical difference between the intercept of MR-Egger and the zero intercept of IVW (*P* = 0.769); hence, there was no horizontal pluripotency. The results of pluripotency analysis between NAFLD and corrected BMI leptin concentration also showed no statistical difference between the intercept of MR-Egger and the zero intercept of IVW. There was no statistical difference (*P* = 0.614); hence, there was no horizontal multi-effectiveness. Another piece of evidence that horizontal multiplicity does not exist is the symmetry of the funnel plot.

**Table 4:** Results of heterogeneity and pluripotency analysis test for data related to leptin and NAFLD

Exposure	Method	Heterogeneity	Pval	Pleiotropy	Pval
		Q value (I2)		Intercept	
Leptin	Inverse variance weighted	3.356201594	0.762992631	− 0.007129678	0.769547522

### Leave-one-out analysis

**Fig. 4:**
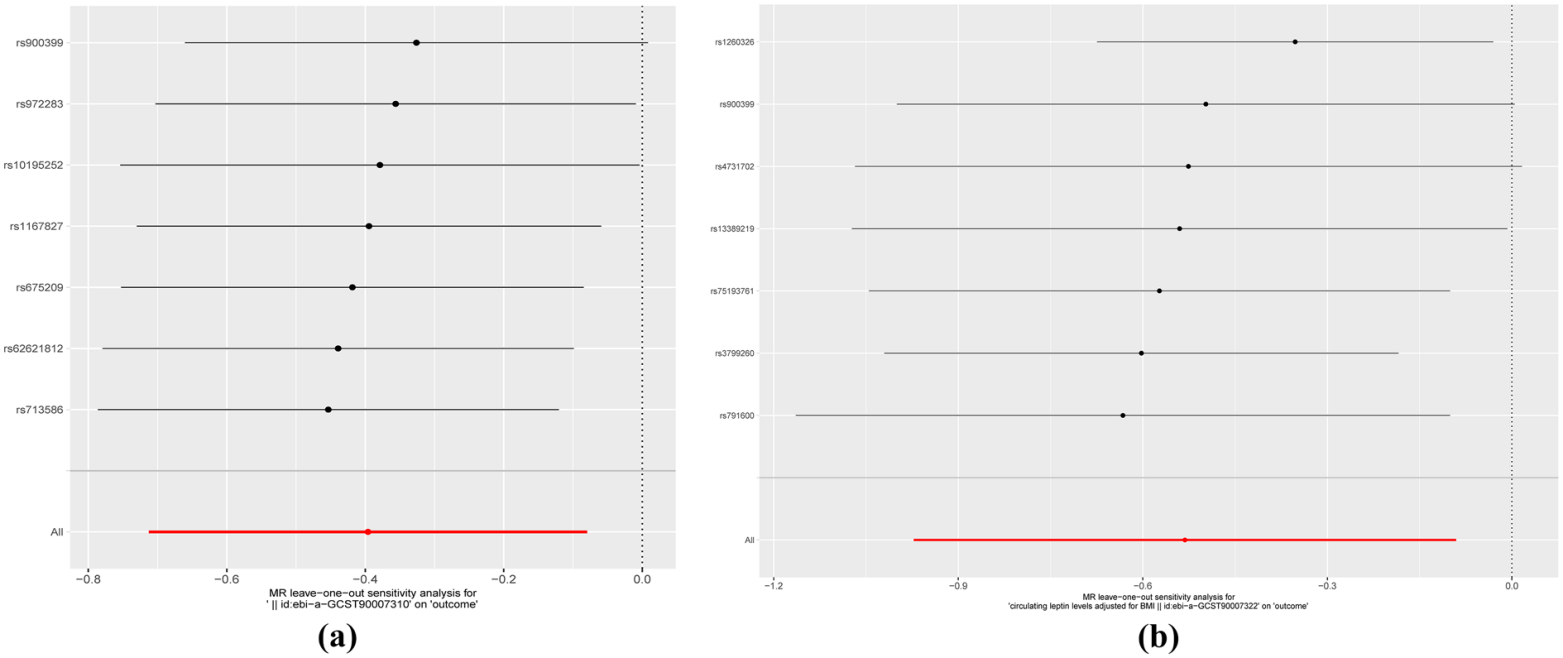
Forest plots for the TSMR leave-one-out analysis of the significant IVW estimates. Within each panel, the black points represent the causal estimate of the association between a specific metabolite and epilepsy after discarding each SNP in turn. Red points represent the pooled IVW estimates. Horizontal lines denote 95% confidence intervals(CI). **a** Leptin; **b** circulating leptin levels adjusted for BMI

Leave-one-out analysis was conducted to calculate the MR result of the remaining IVs after removing them one by one. After excluding each SNP, the overall error line did not change much. The single SNP (rs900399, rs4731702) relatively affects the robustness of the results (Fig. 4). Therefore, the TSMR correlation analysis results of NAFLD with leptin and circulating leptin levels adjusted for BMI were relatively reliable.

## Discussion

In this study, we used TSMR to assess the association between leptin and NAFLD. The OR values obtained using IVW (OR 0.6729; 95% CI 0.4907–0.9235; *P* = 0.0142) and WM methods (OR 0.6549; 95% CI 0.4373–0.9806; *P* = 0.0399) provide strong evidence that elevated levels of leptin are causally associated with a reduced risk of NAFLD, suggesting that leptin may act as a protective factor for NAFLD to some extent. Therefore, even if the exact mechanism is unknown, we believe that increasing the level of leptin in NAFLD patients can lower the risk of NAFLD development. Furthermore, our study initially explored a causal relationship between leptin-related phenotypes and body composition. However, no strong evidence was found to support a causal relationship between leptin and body composition and adipose tissue.

It is worth noting that a group of experts has proposed altering the nomenclature of NAFLD to metabolic-dysfunction-associated fatty liver disease (MAFLD), signaling a shift in the paradigm and underlying etiology toward a more broad term that does not specifically address NAFLD in recent years [48, 49]. Instead of being a disease in and of itself, fatty liver is a histological change in the liver that is a reflection of anomalies in the human metabolic system. As a result, the focus of the disease's treatment is on metabolic management, although in practical practice, there are still a number of bottlenecks to treating NAFLD [9]. To begin with, although there are better recognized and established methods for monitoring NAFLD in traditional high-risk categories (diabetes, hypertension, and overweight), increasing numbers of studies have indicated that NAFLD occurs more commonly in adults with normal BMI (i.e., visceral obesity) [50]. For such patients, especially nonobese people with intermittent transaminase abnormalities, there is still a lack of sufficiently effective biological markers for the diagnosis of NAFLD, as well as a lack of effective techniques for predicting the risk of development. It is due to the fact that liver aspiration biopsy is still a rare procedure, and current guidelines merely prescribe regular follow-up, which does not allow for early treatment [51, 52]. Second, according to the existing guideline recommendations, lifestyle changes, exercise, and diet control are important nonpharmacological options for people with established NAFLD [53]. Still, these nonpharmacological therapies lack sufficient quantifiable indicators, particularly for weight control (guidelines recommend a 5–10% reduction), which is not feasible in lean nonalcoholic fatty liver disease. In people with NAFLD with normal liver enzymes, there is a lack of evaluable serological indicators [54]. Third, due to the complex pathological mechanism of NAFLD itself and the fact that this disease is often only the "tip of the iceberg" of metabolic system diseases, the current treatment of fatty liver lacks sufficiently targeted and recognized effective treatment options. Although, in recent years, there have been advances in treatment options, including the use of a combination of drugs such as semaglutide, firsocostat (ACC inhibitor), and cilofexor (FXR agonist), their reliability and effectiveness need to be confirmed by clinical trials [55]. Therefore, although the treatment of fatty liver is a classic and relatively old topic, developing its specific drugs is still a virgin territory to be explored, and the discovery of more therapeutic targets is of great value for drug development. Based on a review of the literature, leptin regulates food intake, energy balance/body weight, and some metabolic functions [56]. In this regard, leptin should have an anti-steatosis impact on hepatocytes [15]. However, no therapeutics for NAFLD are directed at this target. As a result, the finding that leptin and NAFLD are correlated may be useful for assessing disease risk, preventing NAFLD, combining existing therapy regimens for potentiation, and identifying prospective targets for novel drug development. Our study, based on the literature, did find a significant association between increased leptin levels and reduced incidence of NAFLD, and our findings not only coincide with previous literature but also validate the hypothesis in the literature through database analysis of real-world case sources.

Leptin signals through binding to its receptors, mainly Lep Rb, which is a long stretch of extracellular structure, a transmembrane region and an elongated intracellular extension. As Lep Rb does not possess intrinsic kinase activity, the conformational change of Lep Rb upon leptin binding to Lep Rb induces the activation of Janus kinase (JAK2) phosphorylation, which phosphorylates three tyrosine residues (Y985, Y1077 and Y1138) in the intracellular extension of Lep Rb. These phosphorylated tyrosine residues then recruit proteins containing the SH2 phosphorylation recognition domain for downstream signaling. Currently, the most studied leptin signaling is the JAK/signal transducer and activator of transcription (STAT) pathway. Leptin and NAFLD exert their effects mainly through the JAK2/STAT3 pathway [57, 58]. An important role of leptin is to direct the storage of triglycerides in adipocytes and prevent their deposition in non-adipose tissues such as the liver, thus preventing hepatocyte lipotoxicity and apoptosis. Leptin also inhibits the production of hepatic glucose and the formation of new hepatic fat, acting as an insulin-like agent to prevent the development of NAFLD. Studies have shown that chronic central leptin infusion can reduce hepatic lipid synthesis gene expression and triglyceride levels by stimulating hepatic sympathetic activity and that this effect of leptin is associated with the PI3K signaling pathway, blocking which can specifically induce hepatic steatosis without causing obesity. In addition, leptin promotes fatty acid oxidation in the liver and increases fatty acid consumption in the liver [59]. In addition to its direct effects on the liver, leptin also affects hepatic glucose metabolism indirectly through its central regulation. Leptin infusion into the ventricles of type 1 diabetes mice inhibited the expression of glucagon, consistent with the phenotype of peripheral hyperleptinemia [60]. Specific expression of Lep Rb in the arcuate nucleus of the rat hypothalamus by adenoviral transfection improves peripheral insulin sensitivity and reduces hepatic gluconeogenesis in leptin receptor-deficient Koletsky rats [61]. The regulation of hepatic glucose by leptin may be related to the effect of its phosphatidylinositol 3-kinase (PI3K), which increases insulin signaling and decreases the expression of glucose synthesis genes such as glucose-6-phosphatase (G-6-P) and phosphoenolpyruvate carboxykinase (PEPCK) [61]. In addition, the effects of leptin can also be mediated by central neural regulation, e.g., selective severance of the hepatic vagus nerve can prevent hypothalamic leptin from regulating hepatic insulin sensitivity.

The mechanism of leptin in NAFLD has been supported by a large body of experimental data, and clinical studies on leptin and NAFLD have focused on the association of leptin or leptin receptor levels with NAFLD. The findings on circulating leptin levels in NAFLD patients are not very consistent, with some studies reporting high leptin expression in NAFLD patients [62, 63] and others finding no difference in leptin levels in NAFLD patients compared to non-NAFLD populations [64, 65]. Clinical studies of leptin and Lep R gene expression and SNPs in the NAFLD population have also been reported sporadically. Two small clinical studies showed no expression of the Lep R gene in liver tissue, while in peripheral leukocytes and abdominal adipose tissue Lep gene expression did not differ significantly between NAFLD patients and healthy populations [66, 67]. In another study, immunohistochemical staining of liver tissue for leptin showed that leptin expression was higher in patients with NAFLD than in the healthy population, consistent with altered circulating leptin levels [63]. Some of the Lep R gene SNP studies have also shown a positive association with the development of NAFLD, even if this association is not dependent on the presence of obesity. Given the complexity of clinical studies and the multilevel nature of clinical data, it is difficult to obtain direct evidence that leptin resistance causes NAFLD from the available clinical research data, which need to be interpreted with caution.

Our study has several advantages. First, the TSMR analysis method is based on the principle of Mendelian randomization-free segregation and combination, which excludes the influence of acquired factors (social environment and natural environment) on the study results at the genetic level. In order to successfully compensate for the vulnerability to confounding factors and reverse causality interference in traditional observational studies for inferring the etiology of complex disorders, the genes must arise prior to the disease with a precise causal time sequence [68, 69]. Second, this study uses publicly available GWAS summary statistics with a large sample size to obtain more precise estimates and greater statistical power, saving research costs and improving the utilization of biological information while limiting the study population mainly to individuals of European ancestry, reducing some of the bias that may arise due to population stratification. Finally, the value of this study lies in establishing an association between leptin levels and the incidence of NAFLD using a database of real-world sources. Based on our findings, it is reasonable to believe that leptin levels may be used for the assessment of NAFLD, including the screening of people who are traditionally at high risk of developing NAFLD (e.g., those with comorbid diabetes and those who are overweight), and, more importantly, for the assessment of the risk of developing lean NAFLD in people with normal BMI. In addition, it can be used as an indicator to evaluate the improvement potential of NAFLD. Since there are no drugs that target leptin, we believe that, based on the current state of research, leptin can reduce the incidence of NAFLD without duplicating the mechanism of action of other existing drugs for NAFLD and can be used as a complement to existing treatment regimens. Additionally, there is evidence that leptin regulation may have favorable effects on a variety of other factors, including weight loss, reducing blood sugar levels, and controlling intestinal functions [70]. Therefore, modulation of leptin levels may be used in multiple aspects of metabolic disorders and may have a wider range of potential applications. However, there are some limitations to this study. First and foremost, the majority of these GWAS data are from European populations. It needs to be determined if the findings we described would hold in other people. Second, this study lacks a multidimensional stratification of the heterogeneity of patients with NAFLD. In the future, a multicenter prospective cohort study is needed to fully consider the heterogeneity of NAFLD, integrate demographic characteristics, lifestyle, genetics and other factors to accurately identify high-risk groups for NAFLD, and develop targeted and individualized body mass control strategies, with a view to achieving accurate prevention and control of NAFLD.

## Conclusion

In conclusion, our study reveals a causal relationship between leptin and NAFLD. It thus provides further insight into the factors that may be associated with a reduced risk of NAFLD development. Additionally, from a systems biology standpoint, it aids researchers in better understanding the connections between diverse diseases.

## Data Availability

All the relevant data are provided within the paper and are publicly available.

## Abbreviations

NAFLD: Nonalcoholic fatty liver disease
BMI: Body Mass Index
NASH: Non-alcoholic steatohepatitis
ALT: Alanine transaminase
MR: Mendelian randomization
TSMR: Two-Sample Mendelian Randomization
SNP: Single Nucleotide Polymorphism
GWAS: Genome-Wide Association Studies
IEU: Integrated Epidemiology Unit
IVs: Instrumental variables
IVW: Inverse variance weighted
WM: Weighted Median
OR: Odds Ratio
CI: Confidence interval
LD: Linkage disequilibrium
MAFLD: Metabolic-dysfunction-associated fatty liver disease

## References

[1] Younossi Z, Tacke F, Arrese M, Chander Sharma B, Mostafa I, Bugianesi E, Wai-Sun Wong V, Yilmaz Y, George J, Fan J (2019). Global perspectives on nonalcoholic fatty liver disease and nonalcoholic steatohepatitis. Hepatology.

[2] Younossi ZM, Koenig AB, Abdelatif D, Fazel Y, Henry L, Wymer M (2016). Global epidemiology of nonalcoholic fatty liver disease-meta-analytic assessment of prevalence, incidence, and outcomes. Hepatology.

[3] Yin X, Guo X, Liu Z, Wang J (2023). Advances in the diagnosis and treatment of non-alcoholic fatty liver disease. Int J Mol Sci.

[4] Feldstein AE, Charatcharoenwitthaya P, Treeprasertsuk S, Benson JT, Enders FB, Angulo P (2009). The natural history of non-alcoholic fatty liver disease in children: a follow-up study for up to 20 years. Gut.

[5] Paternostro R, Trauner M (2022). Current treatment of non-alcoholic fatty liver disease. J Intern Med.

[6] Lonardo A, Nascimbeni F, Mantovani A, Targher G (2018). Hypertension, diabetes, atherosclerosis and NASH: Cause or consequence?. J Hepatol.

[7] Neuschwander-Tetri BA (2017). Non-alcoholic fatty liver disease. BMC Med.

[8] Ren Z, Simons P, Wesselius A, Stehouwer CDA, Brouwers M (2023). Relationship between NAFLD and coronary artery disease: a Mendelian randomization study. Hepatology.

[9] Younossi Z, Anstee QM, Marietti M, Hardy T, Henry L, Eslam M, George J, Bugianesi E (2018). Global burden of NAFLD and NASH: trends, predictions, risk factors and prevention. Nat Rev Gastroenterol Hepatol.

[10] Eslam M, George J (2020). Genetic contributions to NAFLD: leveraging shared genetics to uncover systems biology. Nat Rev Gastroenterol Hepatol.

[11] Romeo S, Kozlitina J, Xing C, Pertsemlidis A, Cox D, Pennacchio LA, Boerwinkle E, Cohen JC, Hobbs HH (2008). Genetic variation in PNPLA3 confers susceptibility to nonalcoholic fatty liver disease. Nat Genet.

[12] Jiménez-Cortegana C, García-Galey A, Tami M, Del Pino P, Carmona I, López S, Alba G, Sánchez-Margalet V (2021). Role of leptin in non-alcoholic fatty liver disease. Biomedicines.

[13] Polyzos SA, Kountouras J, Mantzoros CS (2015). Leptin in nonalcoholic fatty liver disease: a narrative review. Metab Clin Exp.

[14] Rotundo L, Persaud A, Feurdean M, Ahlawat S, Kim HS (2018). The Association of leptin with severity of non-alcoholic fatty liver disease: a population-based study. Clin Mol Hepatol.

[15] Polyzos SA, Aronis KN, Kountouras J, Raptis DD, Vasiloglou MF, Mantzoros CS (2016). Circulating leptin in non-alcoholic fatty liver disease: a systematic review and meta-analysis. Diabetologia.

[16] Boutari C, Perakakis N, Mantzoros CS (2018). Association of adipokines with development and progression of nonalcoholic fatty liver disease. Endocrinol Metab.

[17] Adolph TE, Grander C, Grabherr F, Tilg H (2017). Adipokines and non-alcoholic fatty liver disease: multiple interactions. Int J Mol Sci.

[18] Ikejima K, Honda H, Yoshikawa M, Hirose M, Kitamura T, Takei Y, Sato N (2001). Leptin augments inflammatory and profibrogenic responses in the murine liver induced by hepatotoxic chemicals. Hepatology.

[19] Zhang Q, Wang J, Huang F, Yao Y, Xu L (2021). Leptin induces NAFLD progression through infiltrated CD8+ T lymphocytes mediating pyroptotic-like cell death of hepatocytes and macrophages. Digest Liver Dis.

[20] Smith GD, Ebrahim S (2003). 'Mendelian randomization': can genetic epidemiology contribute to understanding environmental determinants of disease?. Int J Epidemiol.

[21] Smith GD, Ebrahim S (2004). Mendelian randomization: prospects, potentials, and limitations. Int J Epidemiol.

[22] Bowden J, Holmes MV (2019). Meta-analysis and Mendelian randomization: a review. Res Synth Methods.

[23] Guo JZ, Xiao Q, Gao S, Li XQ, Wu QJ, Gong TT (2021). Review of mendelian randomization studies on ovarian cancer. Front Oncol.

[24] Davey Smith G, Holmes MV, Davies NM, Ebrahim S (2020). Mendel's laws, Mendelian randomization and causal inference in observational data: substantive and nomenclatural issues. Eur J Epidemiol.

[25] Emdin CA, Khera AV, Kathiresan S (2017). Mendelian randomization. JAMA.

[26] Richmond RC, Davey Smith G (2022). Mendelian randomization: concepts and scope. Cold Spring Harb Perspect Med.

[27] Burgess S, Scott RA, Timpson NJ, Davey Smith G, Thompson SG (2015). Using published data in Mendelian randomization: a blueprint for efficient identification of causal risk factors. Eur J Epidemiol.

[28] Hartwig FP, Davies NM, Hemani G, Davey Smith G (2016). Two-sample Mendelian randomization: avoiding the downsides of a powerful, widely applicable but potentially fallible technique. Int J Epidemiol.

[29] Pierce BL, Ahsan H, Vanderweele TJ (2011). Power and instrument strength requirements for Mendelian randomization studies using multiple genetic variants. Int J Epidemiol.

[30] Hemani G, Zheng J, Elsworth B, Wade KH, Haberland V, Baird D, Laurin C, Burgess S, Bowden J, Langdon R (2018). The MR-Base platform supports systematic causal inference across the human phenome. Elife.

[31] Yaghootkar H, Zhang Y, Spracklen CN, Karaderi T, Huang LO, Bradfield J, Schurmann C, Fine RS, Preuss MH, Kutalik Z (2020). Genetic studies of leptin concentrations implicate leptin in the regulation of early adiposity. Diabetes.

[32] Carter AR, Sanderson E, Hammerton G, Richmond RC, Davey Smith G, Heron J, Taylor AE, Davies NM, Howe LD (2021). Mendelian randomisation for mediation analysis: current methods and challenges for implementation. Eur J Epidemiol.

[33] Ghodsian N, Abner E, Emdin CA, Gobeil É, Taba N, Haas ME, Perrot N, Manikpurage HD, Gagnon É, Bourgault J (2021). Electronic health record-based genome-wide meta-analysis provides insights on the genetic architecture of non-alcoholic fatty liver disease. Cell Rep Med.

[34] van Kippersluis H, Rietveld CA (2018). Pleiotropy-robust Mendelian randomization. Int J Epidemiol.

[35] Chen L, Yang H, Li H, He C, Yang L, Lv G (2022). Insights into modifiable risk factors of cholelithiasis: a Mendelian randomization study. Hepatology.

[36] Davey Smith G, Hemani G (2014). Mendelian randomization: genetic anchors for causal inference in epidemiological studies. Hum Mol Genet.

[37] Yuan S, Chen J, Li X, Fan R, Arsenault B, Gill D, Giovannucci EL, Zheng JS, Larsson SC (2022). Lifestyle and metabolic factors for nonalcoholic fatty liver disease: Mendelian randomization study. Eur J Epidemiol.

[38] Bowden J, Davey Smith G, Haycock PC, Burgess S (2016). Consistent estimation in Mendelian randomization with some invalid instruments using a weighted median estimator. Genet Epidemiol.

[39] Bowden J, Davey Smith G, Burgess S (2015). Mendelian randomization with invalid instruments: effect estimation and bias detection through Egger regression. Int J Epidemiol.

[40] Verbanck M, Chen CY, Neale B, Do R (2018). Detection of widespread horizontal pleiotropy in causal relationships inferred from Mendelian randomization between complex traits and diseases. Nat Genet.

[41] Allen NE, Sudlow C, Peakman T, Collins R (2014). UK biobank data: come and get it. Sci Transl Med.

[42] Burgess S, Thompson SG (2017). Interpreting findings from Mendelian randomization using the MR-Egger method. Eur J Epidemiol.

[43] Huang S, Huang F, Mei C, Tian F, Fan Y, Bao J (2022). Systemic lupus erythematosus and the risk of cardiovascular diseases: a two-sample Mendelian randomization study. Front Cardiovasc Med.

[44] Li Q, Yan S, Li Y, Kang H, Zhu H, Lv C (2022). Mendelian randomization study of heart failure and stroke subtypes. Front Cardiovasc Med.

[45] Bowden J, Del Greco MF, Minelli C, Davey Smith G, Sheehan NA, Thompson JR (2016). Assessing the suitability of summary data for two-sample Mendelian randomization analyses using MR-Egger regression: the role of the I2 statistic. Int J Epidemiol.

[46] Burgess S, Davey Smith G, Davies NM, Dudbridge F, Gill D, Glymour MM, Hartwig FP, Holmes MV, Minelli C, Relton CL (2019). Guidelines for performing Mendelian randomization investigations. Wellcome Open Res.

[47] Burgess S, Thompson SG (2011). Avoiding bias from weak instruments in Mendelian randomization studies. Int J Epidemiol.

[48] Eslam M, Sanyal AJ, George J (2020). MAFLD: a consensus-driven proposed nomenclature for metabolic associated fatty liver disease. Gastroenterology.

[49] Eslam M, Newsome PN, Sarin SK, Anstee QM, Targher G, Romero-Gomez M, Zelber-Sagi S, Wai-Sun Wong V, Dufour JF, Schattenberg JM (2020). A new definition for metabolic dysfunction-associated fatty liver disease: an international expert consensus statement. J Hepatol.

[50] Ismaiel A, Jaaouani A, Leucuta DC, Popa SL, Dumitrascu DL (2021). The visceral adiposity index in non-alcoholic fatty liver disease and liver fibrosis-systematic review and meta-analysis. Biomedicines.

[51] Nassir F (2022). NAFLD: mechanisms, treatments, and biomarkers. Biomolecules.

[52] Wong VW, Adams LA, de Lédinghen V, Wong GL, Sookoian S (2018). Noninvasive biomarkers in NAFLD and NASH - current progress and future promise. Nat Rev Gastroenterol Hepatol.

[53] Younossi ZM (2019). Non-alcoholic fatty liver disease—a global public health perspective. J Hepatol.

[54] Wang AY, Dhaliwal J, Mouzaki M (2019). Lean non-alcoholic fatty liver disease. Clin Nutr.

[55] Mantovani A, Dalbeni A (2021). Treatments for NAFLD: state of art. Int J Mol Sci.

[56] Perakakis N, Farr OM, Mantzoros CS (2021). Leptin in leanness and obesity: JACC state-of-the-art review. J Am Coll Cardiol.

[57] Wauman J, Tavernier J (2011). Leptin receptor signaling: pathways to leptin resistance. Front Biosci.

[58] Myers MG, Leibel RL, Seeley RJ, Schwartz MW (2010). Obesity and leptin resistance: distinguishing cause from effect. Trends Endocrinol Metab.

[59] Lee Y, Yu X, Gonzales F, Mangelsdorf DJ, Wang MY, Richardson C, Witters LA, Unger RH (2002). PPAR alpha is necessary for the lipopenic action of hyperleptinemia on white adipose and liver tissue. Proc Natl Acad Sci USA.

[60] Fujikawa T, Chuang JC, Sakata I, Ramadori G, Coppari R (2010). Leptin therapy improves insulin-deficient type 1 diabetes by CNS-dependent mechanisms in mice. Proc Natl Acad Sci USA.

[61] German J, Kim F, Schwartz GJ, Havel PJ, Rhodes CJ, Schwartz MW, Morton GJ (2009). Hypothalamic leptin signaling regulates hepatic insulin sensitivity via a neurocircuit involving the vagus nerve. Endocrinology.

[62] Lemoine M, Ratziu V, Kim M, Maachi M, Wendum D, Paye F, Bastard JP, Poupon R, Housset C, Capeau J (2009). Serum adipokine levels predictive of liver injury in non-alcoholic fatty liver disease. Liver Int.

[63] Xu D, Huang XD, Yuan JP, Wu J, Fan Y, Luo HS, Yang YH (2011). Impaired activation of phosphatidylinositol 3-kinase by leptin is a novel mechanism of hepatic leptin resistance in NAFLD. Hepatogastroenterology.

[64] Pagano C, Soardo G, Esposito W, Fallo F, Basan L, Donnini D, Federspil G, Sechi LA, Vettor R (2005). Plasma adiponectin is decreased in nonalcoholic fatty liver disease. Eur J Endocrinol.

[65] Kashyap SR, Diab DL, Baker AR, Yerian L, Bajaj H, Gray-McGuire C, Schauer PR, Gupta M, Feldstein AE, Hazen SL (2009). Triglyceride levels and not adipokine concentrations are closely related to severity of nonalcoholic fatty liver disease in an obesity surgery cohort. Obesity.

[66] Chalasani N, Crabb DW, Cummings OW, Kwo PY, Asghar A, Pandya PK, Considine RV (2003). Does leptin play a role in the pathogenesis of human nonalcoholic steatohepatitis?. Am J Gastroenterol.

[67] Cayón A, Crespo J, Mayorga M, Guerra A, Pons-Romero F (2006). Increased expression of Ob-Rb and its relationship with the overexpression of TGF-beta1 and the stage of fibrosis in patients with nonalcoholic steatohepatitis. Liver Int.

[68] Davey Smith G, Ebrahim S (2005). What can mendelian randomisation tell us about modifiable behavioural and environmental exposures?. BMJ.

[69] Tillmann T, Vaucher J, Okbay A, Pikhart H, Peasey A, Kubinova R, Pajak A, Tamosiunas A, Malyutina S, Hartwig FP (2017). Education and coronary heart disease: Mendelian randomisation study. BMJ.

[70] Huang KP, Goodson ML, Vang W, Li H, Page AJ, Raybould HE (2021). Leptin signaling in vagal afferent neurons supports the absorption and storage of nutrients from high-fat diet. Int J Obes.

